# A mobile battery-powered brain perfusion ultrasound (BPU) device designed for prehospital stroke diagnosis: correlation to perfusion MRI in healthy volunteers

**DOI:** 10.1186/s42466-022-00179-8

**Published:** 2022-04-11

**Authors:** Mustafa Kilic, Fabien Scalzo, Chandler Lyle, Dobri Baldaranov, Maximilian Dirnbacher, Tristan Honda, David S. Liebeskind, Felix Schlachetzki

**Affiliations:** 1 Department of Neurology, Center for Vascular Neurology and Intensive Care, University of Regensburg, medbo Bezirksklinikum Regensburg, Universitaetsstr.84, 93053 Regensburg, Germany; 2 Department of Neurology, UCLA Stroke Center and Brain Research Institute, 635 Charles E Young Drive South, Suite 116, Los Angeles, CA 90095 USA; 3 BURL Concepts, Inc., 4901 Morena Boulevard Suite 703, San Diego, CA 92117 USA; 4 Alzheimer’s Therapeutic Research Institute, Keck School of Medicine, University of Southern California, 9860 Mesa Rim Road, San Diego, CA 92121 USA; 5 Department of Neurology, Neurovascular Imaging Research Core and UCLA Stroke Center, University of California Los Angeles, Ronald Reagan UCLA Medical Center, 300 Medical Plaza Driveway B200, Los Angeles, CA 90095 USA

**Keywords:** Ischemic stroke, Large vessel occlusion, Prehospital diagnostics, Brain perfusion ultrasound, Point-of-care ultrasound

## Abstract

**Background:**

Early prehospital stroke identification is crucial for goal directed hospital admission especially in rural areas. However, clinical prehospital stroke scales are designed to identify any stroke but cannot sufficiently differentiate hemorrhagic from ischemic stroke, including large vessel occlusion (LVO) amenable to mechanical thrombectomy. We report on a novel small, portable and battery driven point-of-care ultrasound system (SONAS®) specifically developed for mobile non-invasive brain perfusion ultrasound (BPU) measurement after bolus injection of an echo-enhancing agent suitable for the use in prehospital stroke diagnosis filling a current, unmet and critical need for LVO identification.

**Methods:**

In a phase I study of healthy volunteers we performed comparative perfusion-weighted magnetic resonance imaging (PWI) and BPU measurements, including safety analysis.

**Results:**

Twelve volunteers (n = 7 females, n = 5 males, age ranging between 19 and 55 years) tolerated the measurement extremely well including analysis of blood–brain barrier integrity, and the correlation coefficient between the generated time kinetic curves after contrast agent bolus between PWI and BPU transducers ranged between 0.89 and 0.76.

**Conclusions:**

Mobile BPU using the SONAS® device is feasible and safe with results comparable to PWI. When applied in conjunction with prehospital stroke scales this may lead to a more accurate stroke diagnosis and patients bypassing regular stroke units to comprehensive stroke centers. Further studies are needed in acute stroke patients and in the prehospital phase including assessment of immediate and long-term morbidity and mortality in stroke.

*Trial registration*: Clinical trials.gov, registered 28.Sep.2017, Identifier: NCT03296852.

## Background

Brain perfusion imaging for neurological disorders is currently the domain of perfusion-weighted computed tomography (CTP) and perfusion-weighted Magnetic Resonance Imaging (PWI) [1–6]. The main application in clinical neurology is detection of perfusion deficits in acute stroke especially selecting patients for successful mechanical thrombectomy (MT) and extending the therapeutic window for recanalization therapies up to 24 h. While the number-needed-treat in MT for favorable outcome (defined as modified Rankin Scale 0–2) is 2.3 to 7 is critically dependent on patient selection by cerebral perfusion imaging, successful recanalization and short symptom to recanalization times. The latter is a limiting factor as the prehospital triage as MT is not universally available in stroke units (SU) and secondary transport to comprehensive stroke centers (CSC) are time consuming adding to the still high mortality and morbidity in stroke [7, 8].

Ultrasound is the only modality for widespread brain imaging diagnostics as the mobile SU concept with computed tomography (CT) at the patient’s site are of limited availability and range and the air-mobile SU still in the concept phase [9, 10]. Focusing on the prehospital diagnosis of cerebral artery occlusion and stenosis in acute stroke patients, transcranial color-coded sonography (TCCS) using battery driven color-Duplex point-of care ultrasound (POCUS) system has demonstrated high sensitivity and specificity for the detection of middle cerebral artery occlusion (MCAO) of 90% [11, 12]. However, this technique requires special neurological expertise or extensive training programs for paramedics in this pathophysiological driven diagnostic concept, i.e. focusing on the right middle cerebral artery in left sided hemiparesis and neglect [13].

Only few studies exist focusing on perfusion abnormalities in the context of reperfusion, a putative target for neuroprotective strategies addressing hyperperfusion syndromes and focal brain oedema leading to raised intracranial pressure [14, 15]. Factors limiting the widespread use in screening and follow up of CTP and PWI include the relative high costs and the restrictions regarding the administration of CT or magnetic resonance imaging (MRI) contrast agents, concern of repeated radiation and finally repetitive transport of critically ill patients through the hospital [16–18].

We here present the SONAS® device specifically designed as an easy to use, portable device for non-invasive brain perfusion assessment to aid in early stroke detection and bedside treatment monitoring. In the following, the SONAS® device, its technology and underlying diagnostic concept will be introduced.

## Methods

### Clinical study

The study was performed at the University of California Los Angeles, Department of Neurology, and approved by the local ethics committee (IRB protocol number 16–001,538), in accordance with the WORLD Medical Association Declaration of Helsinki. Since this was a ‘first in human’ study, the intention was to enroll only healthy volunteers with additional study registration at ClinicalTrials.gov (Identifier: NCT03296852). Pre-selection included absence of previous or ongoing cerebrovascular or cardiovascular disease and vascular risk factors, but not presence or absence of sufficient temporal bone windows.

The study aims were to I. determine the safety of the SONAS® device, II. obtain perfusion weighted MRI (PWI) for perfusion quantification in parallel to SONAS® brain perfusion measurements, and III. determine the feasibility to detect microbubble specific frequencies.

### Non-imaging ultrasound perfusion device description

**Fig. 1:**
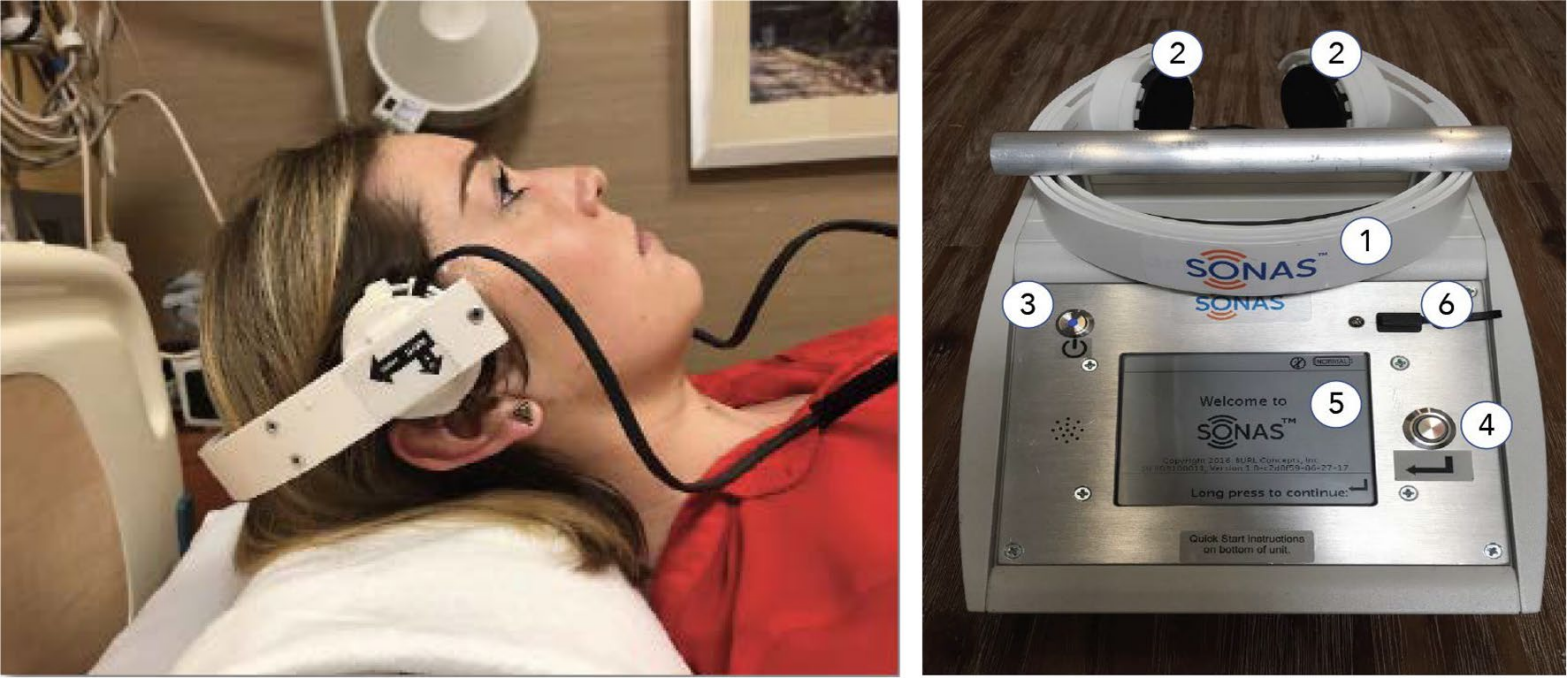
Left: SONAS® headset. Right: SONAS® device, pre-commercial/trial version. Positioning of the transducers above the temporal bone window on both sides of the head with subject in supine position. 1: headset, 2: transducer, 3: power button, 4: “enter” button, 5: LCD screen, 6: USB port. Size: 19 × 19x12cm. Weight: 2,2 kg

The CE-certified (Class IIa), non-imaging ultrasound device SONAS® is portable, has wireless capabilities and is battery-powered to assess brain perfusion in conjunction with ultrasound enhancing agents such as SonoVue® [19]. The pre-commercial version includes an operating unit and a headset with bilateral low frequency transducers for insonation through the temporal bone windows (Fig. 1).

The SONAS® transmit frequency is low for improved temporal bone penetration (220 kHz, 2% duty cycle alternating from right to left, TIC and MI < 1.0). Each transducer assembly contains two individual transducers for transmit and receive in a co-axial configuration. Upon microbubble injection (2.4 ml ultrasound contrast agent SonoVue®) and ultrasound excitation at 220 kHz, the 4th, 5th and 6th harmonic frequencies are used for signal processing. The acquired data sets are separated into two categories: Contralateral (CONTRA) measurements, where the transmission and reception are on opposite sides of the head, and Ipsilateral (IPSI) measurements, where the transmission and reception are on the same side of the head.

SONAS® uses a peak detection algorithm to find the time to peak (TTP) at which the resonant harmonic energy from microbubbles reaches its maximum in each bolus kinetic curve, which are generated after microbubble bolus injection. This is done for both brain hemispheres individually. As a next step, both hemispheric TTP values are compared to each other and the differential is calculated and expressed as a delta-TTP (dTTP) value. Assuming a normal perfusion pattern in both hemispheres the dTTP value is small. In presence of a perfusion deficit, for example due to a stroke, the TTP of the affected hemisphere is delayed, resulting in an increased dTTP value.

### Non-imaging ultrasound and MRI perfusion & blood–brain barrier measurements

Prior to the SONAS® test, volunteers underwent a clinical assessment, followed by a cranial MRI study employing a Siemens 1.5 T Avanto (Erlangen, Germany) equipped with a 12 channel head coil. PWI scanning was performed as previously described [20]. In brief, a timed contrast-bolus passage technique (0.1 mg/kg contrast administered intravenously at a rate of 5 cm3/s) was used with a repetition time (TR) range of 1770 to 2890 ms, and average echo time (TE) of 44 ± 10.4. Pixel dimension varies from 0.859 × 0.859 × 6 to 1.875 × 1.875 × 7 mm. Pre- and post-gadolinium-enhanced T1-weighted images to assess the integrity of the blood–brain barrier (BBB), the latter after SONAS® measurement was also performed as previously published [21]. Conventional T_1_-weighted sequences (TR 548 ms, TE 14 ms) with a slice thickness of 5 mm and a slice gap of 0.32 mm were used. Qualitatively, pre/post contrast-enhanced MRI images were assessed for BBB impairment by an experienced neuro-radiologist (DSL). In addition to the qualitative readings, the same analysis was performed using a commercial MRI software (Perfscape®/Neuroscape®, Olea Medical®, France), developed for automated detection of potential BBB leakage.

**Fig. 2:**
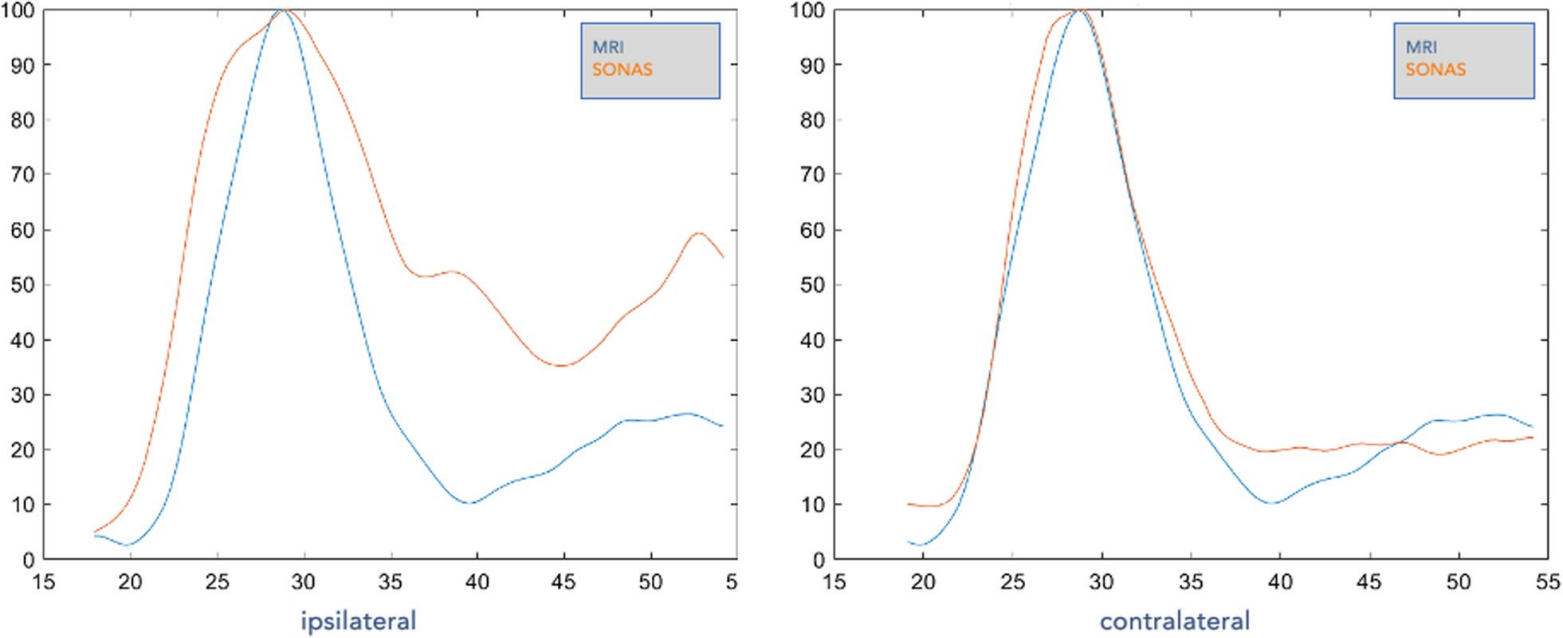
Time intensity curves SONAS® (orange) versus pw-MRI (blue) for both, ipsilateral (left) and contralateral (right) data

After the initial MRI study, the volunteers underwent the SONAS® ultrasound study. The study was performed in supine position. Two ultrasound transducers/probes were positioned on both sides of the volunteer’s head at the temporal bone (above and in front of the ear on each side). To hold the transducers/probes in place a customized headset was designed which was easy to use and comforting for the proband (Fig. 2). An IV line was placed in a cubital or forearm vein. To assess a baseline value, ultrasound was transmitted at an output voltage of 50 V. At this voltage acoustic signals were acquired and stored. The total duration of this first data acquisition was 20 s. For a first SONAS® test (Test 1), a bolus of 2.4 ml of the ultrasound contrast agent (‘Microbubbles’) Lumason™, internationally known as SonoVue® (Bracco Pharmaceuticals, Italy), was injected into the IV line, followed by a 5.0 ml IV bolus injection of saline. Following the IV bolus injection, ultrasound was transmitted, and signals were received. The data acquisition was extended to 40 s. Immediately after the first data acquisition was accomplished, a second SONAS® test (Test 2) was performed in the same fashion to double the total number of data points. Approximately 3–5 h after the SONAS® test the second MRI study (post-contrast T1 sequence to detect BBB leakage) and a second clinical assessment was performed in the same manner. Both assessments (pre/post) were compared to each other and potential deviations assessed.

### Ultrasound perfusion and MRI correlation

The correlation was performed using the Time Intensity Curves (TIC) of all three harmonic frequencies (4th, 5th, 6th) individually as well as their average. Curve smoothing was performed using the Matlab based smoothing algorithm *‘Smoothing Splines’* and the SONAS® peak detection algorithm was applied. Next, the respective MRI TIC’s were generated and the peaks of both sets, SONAS® and MRI, were aligned. The MRI data was then truncated in its beginning and in its ending, achieving a length of 100 samples, like the SONAS® data. Using the Matlab function ‘*corrcoef’*, the correlation coefficient was calculated for both, ipsi- as well as contralateral data.

## Results

In general, a total of n = 12 healthy volunteers (n = 7 Females, n = 5 Males) were enrolled into the study. The age ranged between 19 and 55 years. All participants completed the study. The data of a single volunteer was removed from the data analysis due to technical challenges (thick hair, small skull diameter at the level of the temporal bone) during the SONAS® data acquisition. In this patient both unusual thick hair and a fairly small skull diameter resulted in difficulties to No Adverse Events or Serious Adverse Events occurred.

### Blood–brain barrier (BBB) Integrity

Given the interaction of the ultrasound beam with intravascular microbubbles (i.e. stimulated acoustic emission, harmonic frequency backscatter, cavitation) integrity of the BBB is an essential safety measure. None of the studies showed extravasation of gadolinium into the brain extravascular space excluding measureable BBB leakage, post BPU using the SONAS® device and in comparison, with the corresponding pre SONAS® MRI data.

### TIC Comparison pw-MRI versus SONAS®

TIC's generated by pw-MRI for ipsilateral and contralateral data acquisition were highly comparable (Fig. 2). Two types of TICs were generated for the SONAS data. One was ipsilateral TICs generated by ultrasound transmission as well as signal reception on the same side of the head. The contralateral TICs generated by analyzing the data that resulted from the ultrasound transmission being on one side of the head but the signal reception being on the opposite side of the head. The correlation coefficient between the TICs generated by MRI and the two types of SONAS® TICs was 0.89 for the contralateral and 0.76 for the ipsilateral TICs (Table 1). The difference between these can be explained by acoustic reflection at the skull surface affecting mostly the ipsilateral data acquisition, a phenomenon known from conventional TCCS (so called “near field artifact). In patient #3 and #10 we did find a negative correlation as peak(s) detected in BRU moved in opposite directions in the region of interest compared to PWI most likely due to “noisy”-near field artifacted data failing to produce a proper wash-in wash-out signature. Near field-problems also resulted in inconclusive ipsilateral measurements #2, #4, #6, #7 and #10 while data quality and correlations were generally more robust and higher in the contralateral measurements.

**Table 1:** (a) Ipsilateral (b) Contralateral—average correlation coefficient for each subject and test as well as overall

Subjects	Test	IPSI Left Average Corr. Coeff	IPSI Right Average Corr. Coeff
(a)			
#1	1	0.58	0.93
	2	0.92	0.76
#2	1	–*	0.76
	2	0.98	0.86
#3	1	-0.64	0.96
	2	0.84	0.93
#4	1	0.65	0.79
	2	0.85	–
#5	1	0.71	0.92
	2	0.91	0.67
#6	1	0.97	–
	2	0.99	–
#7	1	0.70	–
	2	–	0.72
#8	1	0.64	0.98
	2	0.87	0.92
#9	1	0.96	0.99
	2	0.97	0.90
#10	1	-0.15	0.99
	2	0.16	–
#11	1	0.95	0.96
	2	0.97	0.88
Overall Average Corr. Coeff		0.76	

Subjects	Test	CONTRA Left–Right Average Corr. Coeff	CONTRA Right-Left Average Corr. Coeff
(b)			
#1	1	0.75	0.83
	2	0.97	0.97
#2	1	0.96	0.97
	2	0.97	0.87
#3	1	0.77	0.92
	2	0.96	0.88
#4	1	0.86	0.93
	2	0.72	0.78
#5	1	0.98	0.94
	2	0.97	0.92
#6	1	0.73	0.84
	2	0.67	0.90
#7	1	0.89	0.76
	2	0.98	0.94
#8	1	0.97	0.97
	2	0.86	0.96
#9	1	0.94	0.93
	2	0.94	0.94
#10	1	0.77	0.98
	2	0.61	0.96
#11	1	0.97	0.96
	2	0.97	0.91
Overall Average Corr. Coeff		0.89	

^*^ ‘–’: signal peak could not be detected due to individual, unfavorable signal-to-noise ratio

## Discussion

This is the first study using a novel portable and battery driven BPU system for measurement of hemispheric brain perfusion and correlation with standard perfusion as determined by PWI. In a first step, we demonstrated a robust correlation of these two methods in healthy volunteers lacking any cerebrovascular pathology. To investigate the potential in stroke diagnosis further studies in patients with acute and chronic brain perfusion alterations, especially acute MCAO are the next step and currently undergoing.

To date, prehospital stroke identification is widely dependent on clinical scales deemed not perfect for detecting LVO leaving decision making based on distances and availability for direct transfer to CSCs instead of regional SU lacking MT capability a complex issue [22–24]. The PRESTO study compared 8 prehospital stroke scales employed by paramedics in the prehospital phase and identified 3 scales suitable to be employed in studies on regional transportation strategies of patients with ischemic stroke including optimization of outcome [25]. An analysis of the Dyon stroke registry applied 16 different prehospital stroke scales (i.e.) prospectively in patients with ischemic stroke and detailed symptom description on first medical examination [26]. Of 971 patients 174 (17.9%) had LVO (defined as MCAO (M1 and M2-segment) and basilar artery), the c-statistic for LVO detection was low (ranging between 0.64 and 0.79), the sensitivity only 59% to 93%, and the specificity ranging from 34 to 89%. The authors conclude, that none of the scales combine a high sensitivity and a high specificity to detect LVO and further studies are needed to determine the best strategy for pre-hospital triage of IS patients [26]. Of note, these results were obtained only in patients with proven ischemic stroke while intracerebral hemorrhage (ICH) were a priori not included which would further weaken the results. In a recent pilot study in the Baltimore metro area demonstrated significantly shorter procedural times for MT of 119 min when re-routing patients for MT to CSCs upon application of the Los Angeles Motor Scale [27]. The significantly faster initiation of MT showed a strong non-significant trend for better outcome in the stroke patients, but also lead to wrong allocation of patients in more than 50%.

Other groups advocate for the implementation of additional stroke diagnostics in the prehospital phase such as blood serum biomarkers and transcranial ultrasound to further streamline diagnostic and therapeutic stroke pathways [28, 29]. Ultrasound diagnostics in this respect are getting widely available due to battery driven small units for POCUS [30, 31]. Hemispheric brain perfusion measurements as being performed by the SONAS® device is a novel technique specifically designed mainly for the prehospital use in stroke. Conventional TCCS identifies MCAO especially after IV echocontrast-enhancing agents (ce-TCCS) in a comparable fashion to CT-Angiography and transcranial perfusion using color-Duplex ultrasound systems and phased-array transducers (pTCS) creating single slice perfusion maps comparable to perfusion-CT [12, 32–34]. However, detailed anatomical knowledge, expertise identifying adequate temporal bone windows and automated perfusion software analysis pose high challenges for both ceTCCS and pTCS. However, part of these obstacles may be overcome using telemetric and artificial intelligence support [35, 36]. The low center transmit frequency of SONAS® device (220 kHz) may allow for scanning even in inferior transcranial bone windows reducing the number of insufficient scans [37]. The presumed high sensitivity of the device for perfusion abnormalities and the user-friendly single button control specifically designed for prehospital use compensate the lack of specific anatomical identification other than hemispheric perfusion.

This study correlates brain perfusion measured by MRI and BPU in healthy volunteers lacking cerebrovascular pathology which per se is a limitation. A large variety of scenarios leading to hemispheric perfusion alteration other than LVO include high grade internal carotid artery stenosis and occlusion, hyperperfusion syndrome as seen after carotid interventions and MT, presence of cerebral arteriovenous malformation, forms of status epilepticus, in hemispheric brain shift, vasospasm and cerebral vasoconstriction syndrome. BPU using SONAS® has a time resolution of 0.4 s compared to 1.8 s in standard MRI suggesting a potential for the detection of more subtle hemispheric brain perfusion differences, but need to be investigated further. Also, other factors such as low cardiac output, aortic valve stenosis, fetal type posterior cerebral arteries or azygos variant of the anterior cerebral artery all of which have an influence on perfusion measurements need to be investigated, and also the quality of the temporal bone window and small/wide skull diameters specifically for BPU. In general, BPU like all techniques in neuroimaging are dependent on a proper clinical question. A limitation for BPU as performed using the SONAS® device is that it is not an imaging device and peripheral arterial vessels close to the convexity, parts of occipital lobes and the cerebellum and brain stem might not be covered by the ultrasound beam area or the signal backscatter not high enough to create a reasonable signal-to-noise ratio. Also, near-field artifacts often limiting measurements in conventional TCCS and TCD also led to inconclusive generation of time-intensity curves, while the contralateral measurements were in general very robust and correlations to PWI good. These issues also need to be addressed in studies in patient with these distinct perfusion alterations and whether or not ipsilateral inconclusive measurements can in future be compensated.

Widespread applications of BPU may include serial inexpensive follow-up cerebral blood flow measurements of patients suffering from Moyamoya disease including acetazolamide testing, assessing brain perfusion abnormalities in forms of dementia, non-invasive measurements of brain perfusion in neurocritical care patients and guiding blood-pressure management in acute intracerebral hemorrhage. A pivotal SONAS® study in acute ischemic stroke including patients suffering from large vessel occlusion prior MT including reperfusion changes such as hyperperfusion after successful MT is currently ongoing. Further refinements, machine learning, advances in transducer technology and artificial intelligence may increase the topical specificity of SONAS®, i.e. perfusion abnormalities in other territories than the MCA [38].

## Conclusion

**Fig. 3:**
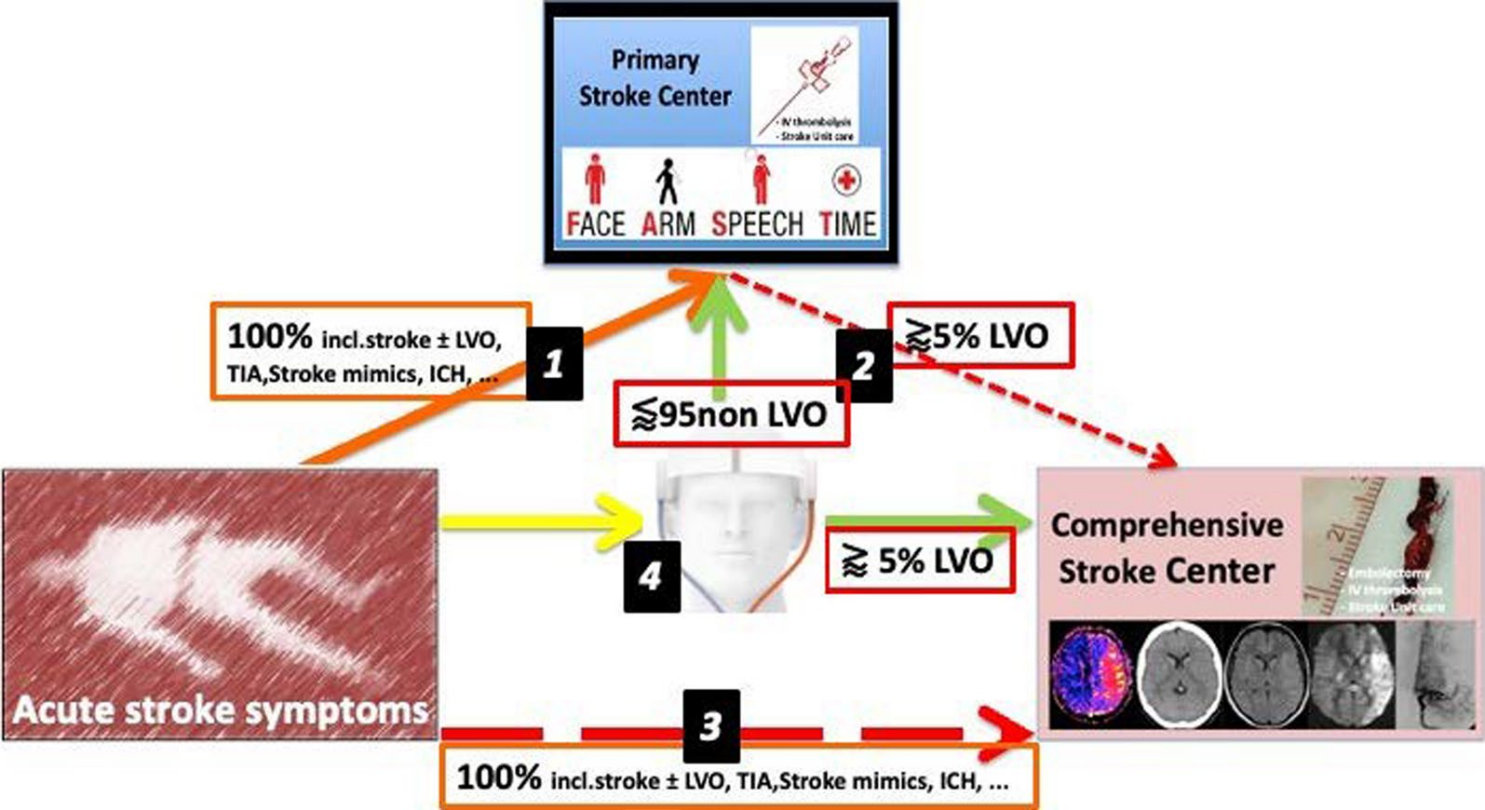
Potential prehospital work flow with or without brain perfusion ultrasound (BPU), numbers are estimates based on all emergency stroke calls, including intracerebral hemorrhage (ICH), stroke mimics, stroke with large vessel occlusion (LVO). (1)—Primary transport of all suspected stroke patients admitted to the next regional stroke unit (rSU): short transport, widely available, fast symptom-to-needle time for thrombolysis, but no mechanical thrombectomy (MT) for LVO available. (2)—Secondary transport from the rSU to comprehensive stroke centers (CSC) for MT resulting in delay in symptom-to-groin time. (3)—Primary transport of all suspected stroke patients to CSCs: Longer symptom-to-needle times, short symptom-to-groin time, but congestion of CSC with non-LVO stroke patients. (4)—Prehospital identification of LVO using BPU and either direct transfer to CSC for MT or to rSU if no perfusion deficit detected

The SONAS® device is able to measure hemispheric brain perfusion thus having the potential to accelerate stroke treatment by identifying LVO in the prehospital phase. This may lead to goal directed hospital admission, fewer secondary transportations, faster symptom to recanalization times, and ultimately better outcome (Fig. 3). Further studies in stroke patients especially with LVO but also intracerebral hemorrhage, lacunar infarction, stroke mimics and minor strokes are needed to define the full potential of this technique.

## Data Availability

The raw data underlying this article are intended for publication on a suitable platform and can be made available by the corresponding author on reasonable request.

## Abbreviations

LVO: Large vessel occlusion
MT: Mechanical thrombectomy
PWI: Perfusion weighted imaging
CTP: Perfusion-weighted computed tomography
SU: Stroke unit
CSC: Comprehensive stroke centers
cCT: Cerebral computed tomography
TCCS: Transcranial color coded sonography
MCAO: Middle cerebral artery occlusion
MRI: Magnetic resonance imaging
CONTRA: Contralateral measurement
IPSI: Ipsilateral measurement
TTP: Time to peak
TIC: Time intensity curve
dTTP: Delta TTP
BBB: Blood–brain barrier
POCUS: Point of care ultrasound
Ce-TCCS: Contrast-enhanced TCCS
pTCS: Perfusion transcranial sonography
